# Changes in kidney functions following acute infusion of low molecular weight polyvinylpyrrolidone in male rats

**DOI:** 10.14814/phy2.70295

**Published:** 2025-03-28

**Authors:** Qi Yan, Qingshang Yan, Henry Shen, Tong Wang

**Affiliations:** ^1^ Department of Cellular and Molecular Physiology, School of Medicine Yale University New Haven Connecticut USA; ^2^ Present address: Department of Biostatistics, College of Public Health University of Kentucky Lexington Kentucky USA

**Keywords:** Na^+^ and K^+^ absorption, PVP infusion, renal clearance

## Abstract

Polyvinylpyrrolidone (PVP), a water‐soluble homopolymer, has been widely used in food, beverage, medical, and experimental tissue preparations. However, the effect of PVP on renal functions remains unknown. We investigated the acute administration of low MW of PVP on renal functions and whether it produces a toxic effect on the kidney. Renal clearance experiments were performed in rats and showed PVP infusion elicited significant diuretic and natriuretic effects. Urine volume, absolute (ENa), and fractional (FENa) Na^+^ excretion were significantly increased. A relatively small kaliuretic effect was also observed. After 2 h of PVP infusion, blood urea nitrogen (BUN) and urinary concentrations of beta‐*N*‐glucosaminidase (NAG) did not change significantly. Alpha‐1‐microglobulin, an indicator of proximal tubule absorption ability, excretion increased 12‐fold, indicating that a large portion of the fluid and Na^+^ loss is due to reduced absorption in the proximal tubule. The 24‐fold increase in ENa and the 12‐fold increase in α1‐microglobulin excretion suggest that fluid and electrolyte absorption were also reduced in other nephron segments. We conclude that acute low molecular weight PVP infusion produces diuretic and natriuretic effects due to the osmotically induced reduction of proximal tubular absorption, and acute PVP infusion does not cause renal damage.

## Graphical abstract

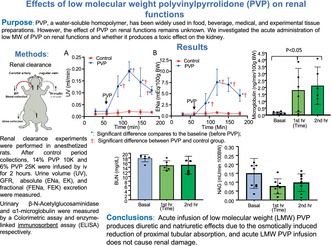

## 1 INTRODUCTION

Polyvinylpyrrolidone (PVP) is the generic name for a water‐soluble homopolymer of *N*‐vinyl‐2‐pyrrolidone (Schwarz, 1990). Invented by Professor Walter Rape in 1939, PVP was initially used as a blood plasma expander in the Second World War (2009; Ravin et al., 1952). However, with relatively inert behavior toward salts and acids and its resistance to thermal degradation in solution (Schwarz, 1990), PVP is now used in more than 100 technical applications (2009) across the pharmaceutical, food, beverage, and cosmetic industries (Nair, 1998). In the United States, PVP is used in many products, including alcohol, water‐based flavored drinks, chewing gum, and tabletop sweeteners (Schwarz, 1990). The synthetic polymer is also used in medical research as a perfusion solution component (Denhardt's solution, some ringer solution) and drug carriers (Kaneda et al., 2004). However, as a macromolecule, PVP is not completely excreted from the body in a short period of time. Approximately, 42.6% and 24.8% of low molecular weight and high molecular weight PVPs were excreted 3 days after a PVP iv infusion (Arisz et al., 1969). The retention and storage of the synthetic polymer persist for at least 20 months after an IV infusion of 35 mg PVP (Altemeier et al., 1954). The kidney is one of the principal storage sites for PVP, and the kidney tubule epithelium was frequently desquamated to form cell casts after seven PVP infusions over 3 weeks (Hartman, 1951). Long‐term local injection and systemic parenteral administration of PVP‐containing solutions caused PVP storage diseases (2009; Altemeier et al., 1954; Arisz et al., 1969; Hartman, 1951; Kaneda et al., 2004; Nair, 1998; Ravin et al., 1952; Schwarz, 1990). Polyvinylpyrrolidone storage disease presents as pathologic fracture and anemia in patients (Leh et al., 2021). Since there is currently no information on the effects of infusion of low molecular weight PVP on kidney function, we investigated whether PVP affects renal functions and whether acute administration of PVP causes renal damage. Renal clearance experiments were performed to measure the urine output (UV), glomerular filtration rate (GFR), absolute Na, K (ENa, EK), and fractional Na, K (FENa, FEK) excretion before and after administration of PVP, and α1‐microglobulin, an indicator of proximal tubule absorption ability, was measured to assess the proximal tubule functions (Yu et al., 1983). To investigate whether acute PVP infusion causes kidney injury, we measured blood urea nitrogen (BUN) and renal tubular damage marker urinary *N*‐acetyl‐β‐d‐glucosaminidase (NAG). BUN is a common marker for evaluating kidney functions (Paget et al., 1958) and the NAG, which is associated with the early detection of atherosclerosis (Kim et al., 2017). Experimental data show PVP did not change the levels of these two markers of BUN or NAG.

## 2 MATERIALS AND METHODS

### 2.1 Animals

Young adult male Sprague–Dawley rats (200–250 g; ~8 weeks old) were purchased from Charles River and housed at the Yale animal facility, allowing free access to food (a standard laboratory rodent diet LAbDiet, from Teklad) and water until the day of the experiment. All procedures for handling and experimenting with rats were approved by the Institutional Animal Use & Care Committee of Yale University. Animals were anesthetized with an intraperitoneal injection (100–150 mg/kg body weight) of Inactin (Thiobutabarbital sodium salt hydrate, Sigma, St. Louis, MO) before the experiment.

### 2.2 Renal clearance and measurements

Renal clearance experiments were performed by using methods developed previously in our laboratory (Yan et al., 2008). Anesthetized rats were placed on a thermostatically controlled surgical table to maintain body temperature at 37°C. After tracheostomy, the left carotid artery was exposed and cannulated with PE 50 polyethylene tubing (Becton Dickinson, New Jersey, NY) for measuring arterial blood pressure and blood collections. The left jugular vein was catheterized with PE10 tubing for intravenous infusion. The bladder was catheterized with PE50 tubing for timed urine collections. After surgical preparation, 0.5 mL of isotonic saline was given intravenously to replace surgical fluid loss. Subsequently, a priming dose of 100 μCi of [methoxy‐3H] inulin (New England Nuclear, Boston, MA) was administered in 0.5mL isotonic saline, and a maintenance infusion of isotonic saline solution containing 10 μCi/mL of inulin at a rate of 4.6 mL/h was provided. An equilibration period of 45 min was followed by two 30‐min collection periods as the controls. After control period collections, 14% PVP 10 K and 6% PVP 25 K (total 20% of PVP) were added to the maintenance infusion during the PVP infusion. The control group was maintained with isotonic saline infusion throughout the experiment. Arterial blood pressure was measured from the cannulated carotid artery by a pressure transducer (Pluznick et al., 2013), and the blood samples were taken in the middle of each urine collection. Urinary and plasma 3H‐inulin counts were measured by Backman counter machine, and urine and plasma Na^+^ and K^+^ concentrations were measured by flame photometry (type 480 Flame Photometer, Corning Medical and Scientific, Corning, NY). Urine volume (UV), glomerular filtration rate (GFR), absolute Na, K (ENa, EK) and fractional Na, K (FENa, FEK) were calculated by standard methods (Yan et al., 2008). PVP 10 K and PVP 25 K (PVP10 and PVP25) were purchased from Sigma‐Aldrich (MilliporeSigma); ^3^H‐Inulin was ordered from New England Nuclear (NEN), Boston, MA.

### 2.3 Enzyme and biochemical measurement

Blood and urine samples, collected from rats before and after the PVP infusion, were stored at −80°C until the time for the measurement. Blood Urea Nitrogen (BUN) reagent set (B7550) and BUN standard (B7550‐STD) were purchased from Pointe Scientific Inc., MI, and the manufacturer's instructions were followed for the sample preparation. Triplicate samples were added to the 96‐well plate and measured by a plate reader at the absorbance wavelength of 340 nm. The concentration of BUN was calculated in milligrams per deciliter (mg/dL). Urinary β‐*N*‐acetylglucosaminidase and α1‐microglobulin were measured by a Colorimetric assay and enzyme‐linked immunosorbent assay (ELISA), respectively. The β‐*N*‐acetylglucosaminidase Assay Kit (CS0780) was purchased from Sigma‐Aldrich, MO; this measurement is based on the hydrolysis of NAG (N‐acetyl‐β‐d‐glucosaminidase) substrate (NP‐GLcNAc) by the enzyme. The enzymatic hydrolysis of the substrate liberates p‐nitrophenylate ion. The reaction product is detected colorimetrically at 405 nm. Rat α1‐microglobulin, α1‐MG ELISA Kit (Catalog #: NB‐E30125) purchased from NovaTeinBio, Inc., MA, was used for the α1‐microglobulin measurements. The protocol of step procedures described in the kit was carefully followed, and reactions were detected by the Infinite M1000 Reader O.D. absorbance wavelength at 450 nm immediately after adding the stop solution.

### 2.4 Statistics

All experimental data are presented as means ± SD. At least six animals were contained in each experimental group. Student's t‐test was used to compare control and experimental groups. One‐way ANOVA test was used to compare multiple experimental groups with a control group followed by Dunnett's test. The difference between the mean values of the experimental and control groups was considered significant if *p* < 0.05.

## 3 RESULTS

### 3.1 Effect of PVP infusion on plasma electrolytes and blood pressure

Table 1 shows the mean blood pressure (BP) and plasma electrolyte before (basal period) and after 1 and 2 h PVP infusion. The BP was kept at the same levels, 127.8, 121.0, and 120.6 mmHg before and after PVP infusion, respectively (*p* > 0.05), indicating the animals were in stable condition during the experimental periods and PVP had no effect on BP. Plasma Na (P_Na_) and plasma K (P_K_) were in the normal range during the control periods as reported previously (Cantone et al., 2008; Yan et al., 2008). PVP infusion did not change P_Na_ or P_K_ (Table 1). The hematocrit was slightly reduced after a 2‐h PVP infusion compared to the basal period (*p* < 0.05). This reduction suggests slight volume expansion due to 2 h iv infusion.

**TABLE 1:** Effect of PVP infusion on blood pressure and plasma electrolyte parameters in rats.

	*N*	BP (mmHg)	PNa (mM)	PK (mM)	HCT%
Basal period	6	127.8 ± 14.9	137.5 ± 9.8	3.50 ± 0.58	36.60 ± 3.13
First hour PVP infusion	6	121.0 ± 17.1	140.3 ± 7.17	3.29 ± 0.39	33.24 ± 3.13
Second hour PVP infusion	6	120.6 ± 17.1	135.5 ± 5.76	3.18 ± 0.32	32.18 ± 0.66*

*Note*: Data are mean ± SD.

Abbreviations: BP, mean arterial blood pressure; HCT, hematocrit; *N*, numbers of the rats; PNa, PK: Plasma Na^+^ and K^+^; PVP, polyvinylpyrrolidone.

* Significantly different from the basal period (*p* < 0.05).

### 3.2 Effect of PVP infusion on renal clearance

**FIGURE 1:**
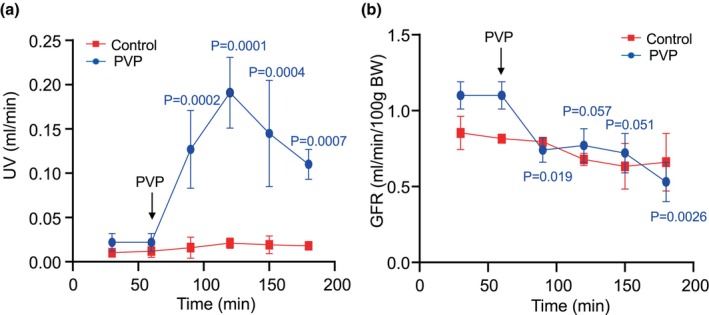
Effect of polyvinylpyrrolidone (PVP) on urine volume (UV) (a) and Glomerular filtration rate (GFR) (b) in rats. After a 60‐min control period, the low molecular weight PVP was given by iv infusion (14% PVP 10 K and 6% PVP 25 K, 4.6 mL/h) for 2 h, and UV and GFR was continuously measured during the PVP infusion (showing in blue). The control group was maintained with isotonic saline infusion throughout the experiment showing in red. Data points are means ± SD. Significant differences are indicated by the *p* volumes.

After two 30‐min control periods of urine collections, the PVP infusion was performed continually for a total of 2 h and additional urine collections were made every 30 min. Urine volume (UV) glomerular filtration rate (GFR), Na^+^, and K^+^ excretion were measured at each collection period (Table 2). Figure 1 summarizes the time course of the effect of PVP on UV (A) and GFR (B). PVP produced a strong diuretic effect, and the peak increase was reached at the 60‐min infusion (showing in blue). As shown in Figure 1a, urine output sharply increased at the first 30 min of infusion and reached its maximum at the second 30‐min period. A significant increase was maintained throughout the experiment, compared with its baseline and with the control group. The PVP infusion increased UV by 4.77 ± 0.82, 7.68 ± 0.77, 5.59 ± 1.04, and 4 ± 0.32 folds, respectively, from the control periods during the 2‐h infusion. UV in the control group, with isotonic saline infusion, was constantly kept at the same level (Figure 1a, showing in red). Figure 1b shows the GFR changes in response to PVP. GFR was reduced by 33% (*P* < 0.05) at the first 30‐min infusion period compared with its baseline and remained almost the same in the subsequent two 30‐min periods of PVP infusion. The reductions of GFR, followed by a strong diuretic effect produced by PVP, were likely due to the increased delivery of fluid and NaCl to the distal nephron, which turned on the TGF (Wright & Schnermann, 1974). There were no significant changes in GFR in the control group (showing in red), indicating that the animals maintained good condition throughout the experiment (Figure 1b).

**TABLE 2:** Effect of PVP on UV, GFR, ENa, EK, FENa, and FEK in rats.

Time (min)	UV (mL/min)	GFR (mL/min)	ENa (μEq/min)	EK (μEq/min)	FENa%	FEK%
Baseline	0.022 ± 0.01	1.10 ± 0.22	0.22 ± 0.20	1.08 ± 0.37	0.12 ± 0.10	28.30 ± 9.99
PVP iv						
0–30	0.127 ± 0.044**	0.74 ± 0.20*	2.86 ± 2.18*	1.87 ± 0.42**	2.93 ± 2.35*	79.82 ± 25.4**
30–60	0.191 ± 0.04**	0.77 ± 0.27	7.58 ± 2.94**	1.88 ± 0.20**	7.33 ± 3.26**	83.18 ± 30.4**
60–90	0.145 ± 0.06**	0.72 ± 0.32	7.04 ± 1.52**	1.81 ± 0.86	7.76 ± 1.44**	81.02 ± 19.2**
90–120	0.110 ± 0.017**	0.53 ± 0.23*	4.59 ± 1.28**	1.16 ± 0.20	7.20 ± 3.03**	75.09 ± 16.7**

*Note*: The values of GFR, ENa, and EK were normalized by 100 g body weight. Data are mean ± SD. *n* = 6 in each group.

Abbreviations: EK, absolute excretion of potassium; ENa, absolute excretion of sodium; FEK, fractional excretion of potassium; FENa, fractional excretion of sodium; GFR, glomerular filtration rate; UV, urine volume.

* Significantly different from baseline control period (*p* < 0.05).

** *p* < 0.01.

**FIGURE 2:**
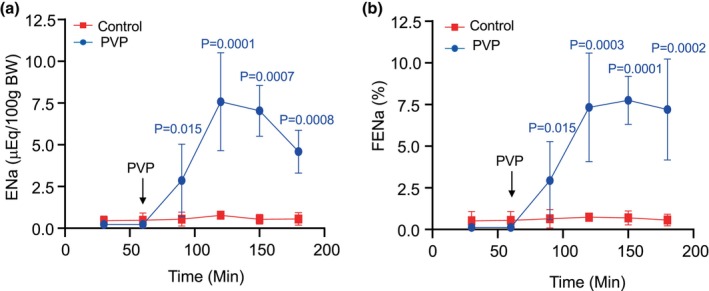
Effect of polyvinylpyrrolidone (PVP) on absolute Na excretion (ENa) (a) and fractional Na excretion (FENa) (b) in rats. After a 60‐min control period, the low molecular weight PVP was given by iv infusion (14% PVP 10 K and 6% PVP 25 K, 4.6 mL/h) for 2 h ENa and FENa were continuously measured during the PVP infusion (showing in blue). The control group was maintained with isotonic saline infusion throughout the experiment showing in red. Data points are means ± SD. Significant differences are indicated by the *p* volumes.

The absolute (ENa) and the fractional sodium excretion (FENa) were measured to evaluate total sodium loss in the urine and the percentage of sodium excreted in the urine versus the sodium reabsorbed by the kidney. As shown in Figure 2, like its strong diuretic effect, PVP also produced a strong natriuretic effect as indicated by the huge increases of both ENa and FENa. ENa was almost tripled after PVP in the first 30‐min period and reached the peak increase at the second 30‐min infusion (Figure 2a), and the significant increments sustained to 2 h. similar to the changes of ENa, FENa was also significantly increased and maintained at a very high level throughout the 2‐h experimental periods (Figure 2b). In contrast, there were no significant changes in ENa and FENa in the control group throughout the experimental period (showing in red).

**FIGURE 3:**
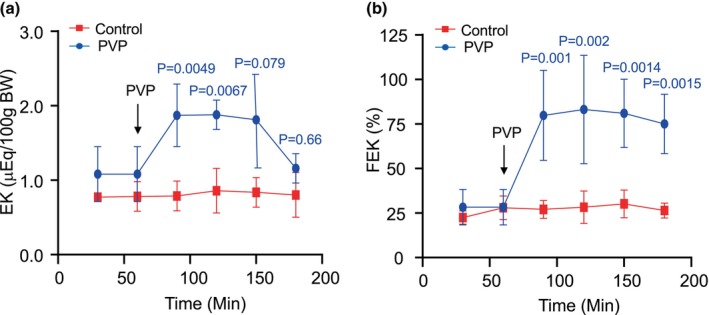
Effect of polyvinylpyrrolidone (PVP) on absolute K excretion (EK) (a) and fractional K excretion (FK) (b) in rats. After a 60‐min control period, the low molecular weight PVP was given by iv infusion (14% PVP 10 K and 6% PVP 25 K, 4.6 mL/h) for 2 h EK and FEK were continuously measured during the PVP in fusion (showing in blue). The control group was maintained with isotonic saline infusion throughout the experiment showing in red. Data points are means ± SD. Significant differences are indicated by the *p* volumes.

**FIGURE 4:**
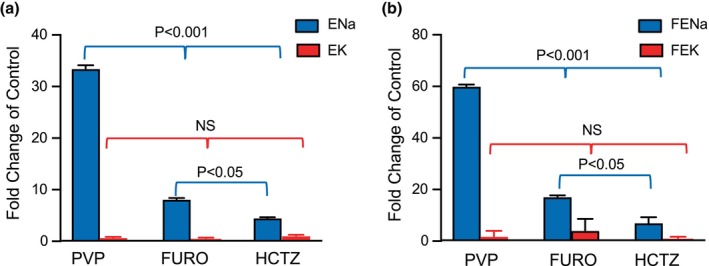
Comparison of effects of polyvinylpyrrolidone (PVP), furosemide (FURO, 8 mg/kg iv), and hydrochlorothiazide (HCTZ, 30 ng/kg iv) on absolute sodium, potassium (ENa, EK) (a) and fractional sodium and potassium (FENa, FEK) (b) excretion. Each bar graph shows the fold changes from the control period, calculated before and after the PVP, FURO, or HCTZ (%). Data points are means ± SD. Significant differences are indicated by the *p* volumes. NS, no significant difference between groups.

The absolute (EK) and the fractional potassium excretion (FEK) were also increased by PVP. EK increased 0.73, 0.74, 0.68, and 0.07‐fold during the four periods of PVP infusion (Figure 3a) and FEK 1.82, 1.94, 1.86, and 1.65, respectively (Figure 3b). PVP produced strong diuretic and natriuretic effects; the kaliuretic effect was much less potent. EK and FEK were also kept at the same level throughout the experimental period in the control group (Figure 3; showing in red). Next, we compared the natriuretic and Kaliuretic effect of pvp with loop diuretic drug furosemide (fouro) and distal tubule diuretic hydrochlorothiazide (HCTZ). Figure 4 shows comparisons of the natriuretic and Kaliuretic effect of PVP, furosemide, and hydrochlorothiazide. The pvp produced a more potent natriuretic effect as indicated by the increments of ENa (Figure 4a), and fractional sodium excretion (FENa) Figure 4b, compared with furo and HCTZ. The increment of ENa and the FENa by PVP was three times higher than furo and HCTZ (*P* < 0.05). In contrast, PVP produced about the same increments of urinary K^+^ excretion with furo and HCTZ, as indicated by both EK and the FEK. This result suggested a different mechanism of PVP‐induced natriuretic effect with furo and HCTZ, likely through inhibition of proximal tubule sodium transport.

### 3.3 Effect of PVP infusion on BUN, urinary excretion of β‐*N*‐Acetylglucosaminidase, and α1‐microglobulin

**FIGURE 5:**
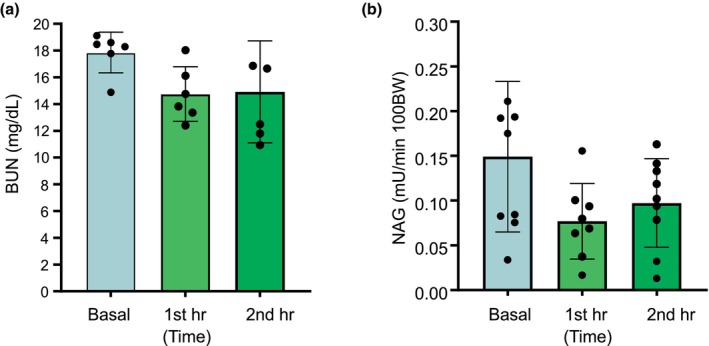
Effect of polyvinylpyrrolidone (PVP) on blood urea nitrogen (BUN) (a) and urinary beta‐*N*‐glucosaminidase (NAG) (b) excretion. BUN and NAG were measured before (Basal) and after the first and second hour PVP infusion. Each data point shows the individual sample measurement, and the bar graph shows the means ± SD. There was no significant difference before and after the PVP infusion.

**FIGURE 6:**
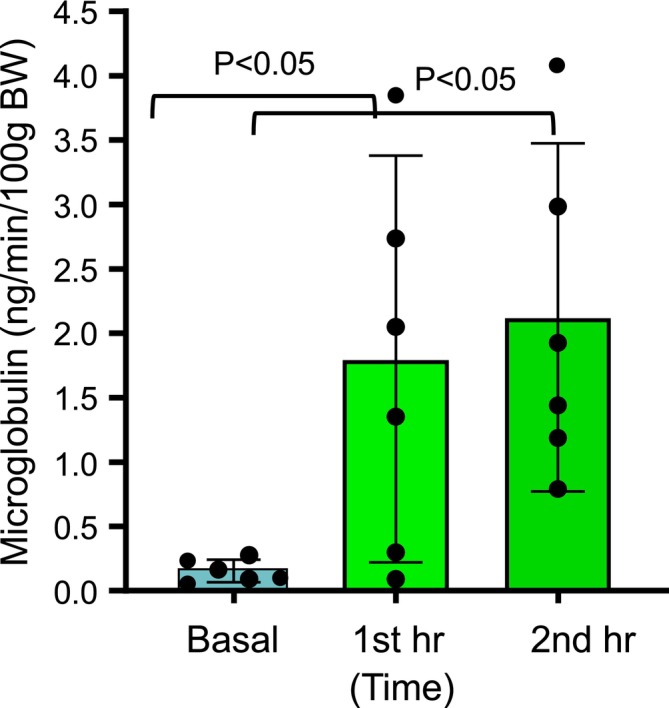
Effect of polyvinylpyrrolidone (PVP) on proximal tubule absorption marker α1‐microglobulin excretion. Urinary excretion of α1‐microglobulin was measured before (Basal) and after the first and second hour PVP infusion. Each data point shows the individual sample measurements, and the bar graph shows the means ± SD. Significant differences are indicated by the *p* volumes.

We examined three indicators to future evaluate kidney functions, to investigate whether infusion of PVP will cause acute kidney injury. the blood urea nitrogen (BUN), urinary concentrations of beta‐*N*‐glucosaminidase (NAG), a lysosomal enzyme involved in the degradation of glycoprotein, and α1‐microglobulin were measured before (basal) and after PVP. As shown in Figure 5a, the BUN kept the same level before and after 1 h and 2 h of infusion of PVP. The NAG was slightly reduced after PVP, but such reductions did not reach statistical significance (*p* > 0.05). However, the α1‐microglobulin, an indicator of proximal tubule absorption function, increased 12‐fold (1.96 vs. 0.15 ng/min/100 g bw) after the PVP (Figure 6), indicating the proximal tubule absorption functions were significantly reduced. There was no evidence of acute kidney or proximal tubule cell injuries since BUN or NAG excretion was not increased.

## 4 DISCUSSION

PVP, a water‐soluble homopolymer of *N*‐vinyl‐2‐pyrrolidone, has been widely used in food, beverage, medical, and experimental tissue preparations (2009; Ravin et al., 1952; Schwarz, 1990). PVP has also been used as a carrier or matrix for drug deliveries and tissue fixations to measure cellular electrolyte concentrations (Siepmann et al., 2010). PVP is a hydrophilic polymer that has been extensively used as a stabilizer in dispersion polymerization to yield microsized polymer particles (Arshady, 1992). However, the effect of PVP on renal functions remains unknown. We investigated the acute administration of PVP on renal functions, including GFR, Na^+^, and K^+^ excretion in rats. We have also investigated whether acute administration of PVP can produce a toxic effect on the kidney. Our experimental results indicated that acute infusion of PVP had no effect on blood pressure but significantly reduced fluid, sodium, and potassium absorption in the kidney, as indicated by the elevated urine volume and urinary Na^+^ and K^+^ excretion. The mechanism of PVP‐produced diuretic and natriuretic effects is different from the classical loop diuretic agent furosemide, which blocks the Na/2Cl/K‐cotransporter (Cantone et al., 2008; Giebisch, 1985), and hydrochlorothiazide, which blocks the thiazide‐sensitive NaCl‐co‐transporter activities (Cantone et al., 2008; Giebisch, 1985). The mechanism of PVP‐reduced Na^+^ absorption is likely due to the inhibition of proximal tubule functions, as indicated by three times more potent natriuretic effects with minimal kaliuretic effect compared with furosemide and hydrochlorothiazide, and the elevation of proximal tubule absorption marker α1‐microglobulin (Yu et al., 1983). In addition, experimental data showed no evidence of kidney injury from acute administration of PVP.

Renal clearance experiments are widely used to measure kidney functions and investigate diuretic agents' efficacy (Giebisch, 1985). Previously by using these methods, we have examined major diuretic agents including furosemide, a well‐known loop diuretic that inhibits NKCC2 (Giebisch, 1985), and hydrochlorothiazide, which blocks the thiazide‐sensitive NaCl‐co transporter function (Giebisch, 1985). Na/2Cl/K‐cotransporter (NKCC2) is the major ion transporter expressed in the apical membrane of the thick ascending limb (TAL) and is responsible for the absorption of an extensive portion of filtered NaCl in the loop of Henle (Hebert & Gullans, 1995). The loop of Henle contributes to the absorption of approximately 25% of filtered sodium and can be targeted by diuretic therapy such as furosemide (Giebisch et al., 1993). Mutations in NKCC2 cause Bartter's syndrome with hypokalemic alkalosis and hypercalciuria (Wagner et al., 2008). Inhibition of NKCC2 by furosemide produces significant diuretic and natriuretic effects and increases urinary K^+^ excretion (Cantone et al., 2008; Giebisch, 1985). The thiazide‐sensitive Na–Cl cotransporter (TSC or NCC) is expressed on the apical membranes of distal convoluted tubule epithelial cells (Plotkin et al., 1996). NCC is the major ion transporter in the distal convoluted tubule (DCT) and is responsible for filtrate Na^+^ and Cl^−^ reabsorption in the distal nephron (Simon & Lifton, 1998). Mutations of the NCC cause Gitelman's syndrome (Simon & Lifton, 1996). NCC is also a major target for the classical diuretic agent hydrochlorothiazide (HCTZ). Using the renal clearance method, we compared the PVP‐induced diuretic and natriuretic effects with the loop diuretic furosemide and the NCC inhibitor HCTZ. Our experimental data show that PVP produced more potent diuretic and natriuretic effects but with the same kaliuretic effect as the loop diuretic and HCTZ (Figure 4). These results suggested a different mechanism involved in the PVP‐induced renal function changes.

Twenty percent PVP was infused by iv at the rate of 4.6 mL/h to get a concentration of about 1.82%–1.54% (calculated based on the 30% body weight of ECF) in the body after 1 h of infusion. This concentration is close to what was reported in clinical use (Ravin et al., 1952) and the micropuncture study (Persson et al., 1972). Since the low molecular weight of PVP can pass rapidly through the glomerulus into proximal tubules and be eliminated through the kidneys (Schwarz, 1990), we examined whether PVP infusion affects proximal tubule transport. α1‐microglobulin is a low‐molecular‐weight protein of 26 kDa and a member of the lipocalin protein superfamily (Larsson et al., 2004). It is synthesized in the liver, freely filtered by glomeruli, and reabsorbed by renal proximal tubule cells, where it is catabolized (Kristiansson et al., 2020). We have measured urinary α1‐microglobulin excretion and found that PVP significantly increased α1‐microglobulin excretion. This result indicated that proximal tubule absorption ability was reduced (Bernard et al., 1987). A direct measurement of the effect of PVP on proximal tubule transport has been done previously (Persson et al., 1972). The net fluid absorption was measured by a split‐oil‐drop micropuncture experiment in proximal tubules of rat kidneys. It has shown that applications of 4%–7% PVP in the tubule significantly reduced fluid outflux, which demonstrated that PVP inhibited fluid absorption in the proximal tubule (Persson et al., 1972). Our result is consistent with their finding.

The major mechanism of PVP‐induced diuretic and natriuretic effects is likely via reduced proximal tubule absorption caused by a volume expansion by PVP infusion, the same effect produced by mannitol (Dry, 1963; Sonnenberg & Solomon, 1969). The osmotic diuretics have a major impact on the proximal convoluted tubule and the descending limb of the loop of Henle. It inhibits water reabsorption in the proximal convoluted tubule and the thin descending loop of Henle and collecting duct, regions of the kidney that are highly permeable to water. The presence of a nonabsorbable solute, such as mannitol, prevents the normal absorption of water by interposing a countervailing osmotic force; as a result, urine volume increases (Dry, 1963; Sonnenberg & Solomon, 1969). Our data showed that a 24‐fold increase in ENa and only a 12‐fold increase in α1‐microglobulin excretion suggest that fluid and electrolyte absorption were also reduced in other nephron segments, such as in the loop of Henle. In addition, PVP infusion also produced a significant increase in urinary K^+^ excretion; the mechanism may be due to a reduction in K^+^ absorption in the proximal tubule and increased K^+^ secretion in the distal nephron. Reductions in proximal tubule absorption of fluid and Na^+^ (Persson et al., 1972) will increase distal delivery of Na^+^ and stimulate distal Na^+^ absorption, which will make the luminal potential more negative and thus increase K^+^ secretion (Palmer, 2015; Wang & Giebisch, 2009).

To investigate whether an acute infusion of PVP causes tubule cell damage, we further examined the effect of PVP on another tubule damage marker, *N*‐acetyl‐β‐d‐glucosaminidase (NAG) excretion. The NAG is a lysosomal enzyme found predominantly in proximal tubules (D'Amico & Bazzi, 2003) and is released from proximal tubule epithelial cells due to acute kidney injury (AKI). The increase of NAG is strongly correlated with the extent of proximal tubule damage (Skalova, 2005). In a comparison of several tubule injury markers, it was demonstrated that increased amounts of NAG are the most common enzymes released by the brush‐border of the proximal tubule cells. This enzyme was used to detect early‐stage and acute damage of epithelial cells (D'Amico & Bazzi, 2003). Therefore, we measured the urinary NAG excretion before and after the infusion of PVP. We found no change in this enzyme, which suggested that PVP did not induce acute renal tubule damage.

PVP‐induced kidney toxicity is dependent on its molecular weight since the permeability and elimination of PVP were inversely related to its molecular weight (MV). Low molecular weight PVP (<25,000) can pass through glomerular capillaries, but higher molecular weight PVP goes directly from postglomerular capillaries into interstices or lymphatic channels without passing the ultrafiltrate (Chi et al., 2006; Stalund et al., 2022). PVP with a MW <20,000 established equilibrium within an hour, and 40%–80% of infused PVP was recovered in the urine within 48 h. At 15 and 30 min after PVP infusion, a high level of PVP was detected in the blood, kidneys, urinary bladder, lungs, skin and hair follicles, sclera, connective tissue, interstitial tissue, and tissue spaces. At 8 h, only the kidneys, areas of the lungs, and intestinal contents had high levels of PVP. Low molecular weight PVP passes rapidly through the glomerulus and is eliminated through the kidneys. The T 1/2 for the elimination of PVP in the low molecular weight has been reported to be as low as 12 h and as high as 72 h (Chi et al., 2006; Gartner et al., 1968). However, larger molecular particles can be retained in the monocyte/macrophage system. Since there was no metabolic degradation of PVP and no significant route of excretion except by the kidneys (Ravin et al., 1952), 35%–49% of PVP is retained indefinitely in the body after iv infusion (Altemeier et al., 1953). Retention of PVP showed slight enlargement of the spleen, damaged the liver and kidneys, some organs became granules and vacuoles, and some nephron tubular epithelium formed casts (Hartman, 1951). Our current data suggested that the low MW weight PVP by acute use does not cause renal injury.

There are several limitations of the work. First, only male rats were used in this study. It is well established that there are sex differences in the regulation of kidney functions, including in our previously published work (Li et al., 2019; Xu et al., 2021; Yan et al., 2008). Whether the response of PVP‐induced changes in renal functions is different between males and females is unknown. Second, the experiments do not define if the effects of PVP are only at the PT, and whether effects on distal segments might contribute to the strong natriuresis, with other effects perhaps exerting anti‐kaliuretic effects (e.g., by inhibition of ENaC, ROMK, or BK channels). Lithium clearance might also be informative since osmotic diuretics increase it, whereas loop and DCT diuretics lower it (Koomans et al., 1989; Skøtt, 1994; Stokke et al., 1990). Direct comparison with mannitol might also be performed. Third, the kidney tissue was not collected to measure ion transporters (such as NHE3, NCC, NKCC2, ENaC, or ROMK) trafficking and expression, and other parameters such as calcium, magnesium, and bicarbonate that might be affected by the PVP were not examined.

In summary, we have demonstrated that acute administration of low molecular weight PVP produces significant diuretic, natriuretic, and kaliuretic effects. The effects differ from the classical loop diuretic furosemide and DCT transport inhibitor hydrochlorothiazide. Acute use of low molecular weight PVP reduces proximal tubule transport function but does not cause significant renal or tubule cell damage.

## Data Availability

The data supporting the findings of this study are available from the corresponding author upon request.

## References

[phy270295-bib-0002] Altemeier, W. A. , Schiff, L. , Gall, E. A. , Giuseffi, J. , Freiman, D. , Mindrum, G. , & Braunstein, H. (1954). Physiological and pathological effects of long‐term polyvinylpyrrolidone retention. A.M.A. Archives of Surgery, 69(3), 309–314. 13188506

[phy270295-bib-0003] Altemeier, W. A. , Schiff, L. , Gall, E. A. , Giuseffi, J. , Hamilton, D. , Freiman, D. , & Braunstein, H. (1953). Long‐term studies on the effect of polyvinylpyrrolidone retention in human patients. Surgical Forum, 4, 724–730. 13187374

[phy270295-bib-0004] Arisz, L. , Hazenberg, B. P. , van Zanten, A. , & Mandema, E. (1969). Renal excretion of low and high molecular weight polyvinylpyrrolidone (Pvp) in patients with proteinuria. Acta Medica Scandinavica, 186(1–6), 393–400. 5378339 10.1111/j.0954-6820.1969.tb01492.x

[phy270295-bib-0005] Arshady, R. (1992). Suspension, emulsion, and dispersion polymerization: A methodological survey. Colloid and Polymer Science, 270(8), 717–732.

[phy270295-bib-0006] Bernard, A. M. , Vyskocil, A. A. , Mahieu, P. , & Lauwerys, R. R. (1987). Assessment of urinary retinol‐binding protein as an index of proximal tubular injury. Clinical Chemistry, 33(6), 775–779. 3297418

[phy270295-bib-0007] Cantone, A. , Yang, X. , Yan, Q. , Giebisch, G. , Hebert, S. C. , & Wang, T. (2008). Mouse model of type II Bartter's syndrome. I. Upregulation of thiazide‐sensitive Na‐Cl cotransport activity. American Journal of Physiology. Renal Physiology, 294(6), F1366–F1372. 18385266 10.1152/ajprenal.00608.2007

[phy270295-bib-0008] Chi, C. C. , Wang, S. H. , & Kuo, T. T. (2006). Localized cutaneous polyvinylpyrrolidone storage disease mimicking cheilitis granulomatosa. Journal of Cutaneous Pathology, 33(6), 454–457. 16776723 10.1111/j.0303-6987.2006.00476.x

[phy270295-bib-0009] D'Amico, G. , & Bazzi, C. (2003). Urinary protein and enzyme excretion as markers of tubular damage. Current Opinion in Nephrology and Hypertension, 12(6), 639–643. 14564202 10.1097/01.mnh.0000098771.18213.a6

[phy270295-bib-0010] Dry, J. (1963). Mannitol (“osmotic diuretic”) in the preventive and curative treatment of acute renal insufficiency. Le Progrés Médical, 91, 717. 14093272

[phy270295-bib-0001] Fisher S. , & Bauer S. (2009). Polyvinylpyrrolidon. Ein Tausendsassa in der Chemie. Chemie in Unserer Zeit, 43(6), 365–439.

[phy270295-bib-0011] Gartner, K. , Vogel, G. , & Ulbrich, M. (1968). Studies on the penetration of macromolecules (polyvinylpyrrolidone) through glomerular and postglomerular capillaries into the urine and kidney lymph, and on the extent of extravascular circulation of 131‐I‐albumin in the kidney interstitium. Pflügers Archiv für die Gesamte Physiologie des Menschen und der Tiere, 298(4), 305–321. 5240012

[phy270295-bib-0012] Giebisch, G. (1985). The use of a diuretic agent as a probe to investigate site and mechanism of ion transport processes. Arzneimittel‐Forschung, 35(1A), 336–342. 2580541

[phy270295-bib-0013] Giebisch, G. , Klein‐Robbenhaar, G. , Klein‐Robbenhaar, J. , Ratheiser, K. , & Unwin, R. (1993). Renal and extrarenal sites of action of diuretics. Cardiovascular Drugs and Therapy, 7(Suppl 1), 11–21. 8435373 10.1007/BF00877954

[phy270295-bib-0014] Hartman, F. W. (1951). Tissue changes following the use of plasma substitutes. A.M.A. Archives of Surgery, 63(6), 728–738. 14868240 10.1001/archsurg.1951.01250040744003

[phy270295-bib-0015] Hebert, S. C. , & Gullans, S. R. (1995). The electroneutral sodium‐(potassium)‐chloride co‐transporter family: A journey from fish to the renal co‐transporters. Current Opinion in Nephrology and Hypertension, 4(5), 389–391. 8564439 10.1097/00041552-199509000-00002

[phy270295-bib-0016] Kaneda, Y. , Tsutsumi, Y. , Yoshioka, Y. , Kamada, H. , Yamamoto, Y. , Kodaira, H. , Tsunoda, S. I. , Okamoto, T. , Mukai, Y. , Shibata, H. , Nakagawa, S. , & Mayumi, T. (2004). The use of PVP as a polymeric carrier to improve the plasma half‐life of drugs. Biomaterials, 25(16), 3259–3266. 14980420 10.1016/j.biomaterials.2003.10.003

[phy270295-bib-0017] Kim, S. R. , Lee, Y.‐h. , Lee, S.‐G. , Kang, E. S. , Cha, B.‐S. , & Lee, B.‐W. (2017). The renal tubular damage marker urinary *N*‐acetyl‐β‐d‐glucosaminidase may be more closely associated with early detection of atherosclerosis than the glomerular damage marker albuminuria in patients with type 2 diabetes. Cardiovascular Diabetology, 16(1), 16. 28122570 10.1186/s12933-017-0497-7 PMC5267389

[phy270295-bib-0018] Koomans, H. A. , Boer, W. H. , & Dorhout Mees, E. J. (1989). Evaluation of lithium clearance as a marker of proximal tubule sodium handling. Kidney International, 36(1), 2–12. 2681925 10.1038/ki.1989.153

[phy270295-bib-0019] Kristiansson, A. , Davidsson, S. , Johansson, M. E. , Piel, S. , Elmér, E. , Hansson, M. J. , et al. (2020). α(1)‐microglobulin (A1M) protects human proximal tubule epithelial cells from heme‐induced damage in vitro. International Journal of Molecular Sciences, 21(16), 5825. 32823731 10.3390/ijms21165825 PMC7461577

[phy270295-bib-0020] Larsson, J. , Allhorn, M. , & Kerstrom, B. (2004). The lipocalin alpha(1)‐microglobulin binds heme in different species. Archives of Biochemistry and Biophysics, 432(2), 196–204. 15542058 10.1016/j.abb.2004.09.021

[phy270295-bib-0021] Leh, F. , Stalund, I. V. , Bjånes, T. K. , Ohldieck, C. , Svarstad, E. , & Leh, S. (2021). Polyvinylpyrrolidone deposition disease in patients with intravenous opioid use: A case series. Human Pathology, 116, 102–111. 34329652 10.1016/j.humpath.2021.07.009

[phy270295-bib-0022] Li, J. , Xu, S. , Yang, L. , Yang, J. , Wang, C. J. , Weinstein, A. M. , et al. (2019). Sex difference in kidney electrolyte transport II: Impact of K(+) intake on thiazide‐sensitive cation excretion in male and female mice. American Journal of Physiology. Renal Physiology, 317(4), F967–F977. 31390232 10.1152/ajprenal.00125.2019 PMC6843050

[phy270295-bib-0023] Nair, B. (1998). Final report on the safety assessment of polyvinylpyrrolidone (PVP). International Journal of Toxicology, 17(4_suppl), 95–130.

[phy270295-bib-0024] Paget, M. , Langeron, L. , Liefooghe, J. , & Nolf, V. (1958). Blood nitrogen levels & urea clearance in kidney function tests. Annales de Biologie Clinique, 16(5–6), 374–375. 13545620

[phy270295-bib-0025] Palmer, B. F. (2015). Regulation of potassium homeostasis. Clinical Journal of the American Society of Nephrology, 10(6), 1050–1060. 24721891 10.2215/CJN.08580813 PMC4455213

[phy270295-bib-0026] Persson, A. E. G. , Ågerup, B. , & Schnermann, J. (1972). The effect of luminal application of colloids on rat proximal tubular net fluid flux. Kidney International, 2(4), 203–213. 4657921 10.1038/ki.1972.96

[phy270295-bib-0027] Plotkin, M. D. , Kaplan, M. R. , Verlander, J. W. , Lee, W. S. , Brown, D. , Poch, E. , Gullans, S. R. , & Hebert, S. C. (1996). Localization of the thiazide sensitive Na‐Cl cotransporter, rTSC1 in the rat kidney. Kidney International, 50(1), 174–183. 8807586 10.1038/ki.1996.300

[phy270295-bib-0028] Pluznick, J. L. , Protzko, R. J. , Gevorgyan, H. , Peterlin, Z. , Sipos, A. , Han, J. , Brunet, I. , Wan, L. X. , Rey, F. , Wang, T. , Firestein, S. J. , Yanagisawa, M. , Gordon, J. I. , Eichmann, A. , Peti‐Peterdi, J. , & Caplan, M. J. (2013). Olfactory receptor responding to gut microbiota‐derived signals plays a role in renin secretion and blood pressure regulation. Proceedings of the National Academy of Sciences of the United States of America, 110(11), 4410–4415. 23401498 10.1073/pnas.1215927110 PMC3600440

[phy270295-bib-0029] Ravin, H. A. , Seligman, A. M. , & Fine, J. (1952). Polyvinyl pyrrolidone as a plasma expander; studies on its excretion, distribution and metabolism. The New England Journal of Medicine, 247(24), 921–929. 13002648 10.1056/NEJM195212112472403

[phy270295-bib-0030] Schwarz, W. (1990). A critical review of the kinetics and toxicology of polyvinylpyrrolidone (povidone) (1st ed.), B. V. Robinson by Leqis Publishers.

[phy270295-bib-0031] Siepmann, F. , Eckart, K. , Maschke, A. , Kolter, K. , & Siepmann, J. (2010). Modeling drug release from PVAc/PVP matrix tablets. Journal of Controlled Release, 141(2), 216–222. 19737588 10.1016/j.jconrel.2009.08.027

[phy270295-bib-0032] Simon, D. B. , & Lifton, R. P. (1996). The molecular basis of inherited hypokalemic alkalosis: Bartter's and Gitelman's syndromes. The American Journal of Physiology, 271(5 Pt 2), F961–F966. 8945989 10.1152/ajprenal.1996.271.5.F961

[phy270295-bib-0033] Simon, D. B. , & Lifton, R. P. (1998). Mutations in Na(K)Cl transporters in Gitelman's and Bartter's syndromes. Current Opinion in Cell Biology, 10(4), 450–454. 9719864 10.1016/s0955-0674(98)80057-4

[phy270295-bib-0034] Skalova, S. (2005). The diagnostic role of urinary *N*‐acetyl‐beta‐d‐glucosaminidase (NAG) activity in the detection of renal tubular impairment. Acta Medica (Hradec Králové), 48(2), 75–80. 16259316

[phy270295-bib-0035] Skøtt, P. (1994). Lithium clearance in the evaluation of segmental renal tubular reabsorption of sodium and water in diabetes mellitus. Danish Medical Bulletin, 41(1), 23–37. 8187564

[phy270295-bib-0036] Sonnenberg, H. , & Solomon, S. (1969). Mechanism of natriuresis following intravascular and extracelluar volume expansion. Canadian Journal of Physiology and Pharmacology, 47(2), 153–159. 5783156 10.1139/y69-026

[phy270295-bib-0037] Stalund, I. V. , Grønseth, H. , Reinholt, F. P. , Svarstad, E. , Marti, H. P. , & Leh, S. (2022). Chronic kidney disease from polyvinylpyrrolidone deposition in persons with intravenous drug use. Clinical Journal of the American Society of Nephrology, 17(4), 518–526. 35296512 10.2215/CJN.13681021 PMC8993479

[phy270295-bib-0038] Stokke, E. S. , Ostensen, J. , Hartmann, A. , & Kiil, F. (1990). Loop diuretics reduce lithium reabsorption without affecting bicarbonate and phosphate reabsorption. Acta Physiologica Scandinavica, 140(1), 111–118. 2125801 10.1111/j.1748-1716.1990.tb08981.x

[phy270295-bib-0039] Wagner, C. A. , Loffing‐Cueni, D. , Yan, Q. , Schulz, N. , Fakitsas, P. , Carrel, M. , Wang, T. , Verrey, F. , Geibel, J. P. , Giebisch, G. , Hebert, S. C. , & Loffing, J. (2008). Mouse model of type II Bartter's syndrome. II. Altered expression of renal sodium‐ and water‐transporting proteins. American Journal of Physiology. Renal Physiology, 294(6), F1373–F1380. 18322017 10.1152/ajprenal.00613.2007

[phy270295-bib-0040] Wang, W. H. , & Giebisch, G. (2009). Regulation of potassium (K) handling in the renal collecting duct. Pflügers Archiv / European Journal of Physiology, 458(1), 157–168. 18839206 10.1007/s00424-008-0593-3 PMC2730119

[phy270295-bib-0041] Wright, F. S. , & Schnermann, J. (1974). Interference with feedback control of glomerular filtration rate by furosemide, triflocin, and cyanide. The Journal of Clinical Investigation, 53(6), 1695–1708. 4830232 10.1172/JCI107721 PMC302666

[phy270295-bib-0042] Xu, S. , Li, J. , Yang, L. , Wang, C. J. , Liu, T. , Weinstein, A. M. , Palmer, L. G. , & Wang, T. (2021). Sex difference in kidney electrolyte transport III: Impact of low K intake on thiazide‐sensitive cation excretion in male and female mice. Pflügers Archiv, 473(11), 1749–1760. 34455480 10.1007/s00424-021-02611-5 PMC8528772

[phy270295-bib-0043] Yan, Q. , Yang, X. , Cantone, A. , Giebisch, G. , Hebert, S. , & Wang, T. (2008). Female ROMK null mice manifest more severe Bartter II phenotype on renal function and higher PGE2 production. American Journal of Physiology. Regulatory, Integrative and Comparative Physiology, 295(3), R997–R1004. 18579648 10.1152/ajpregu.00051.2007 PMC2536865

[phy270295-bib-0044] Yu, H. , Yanagisawa, Y. , Forbes, M. A. , Cooper, E. H. , Crockson, R. A. , & MacLennan, I. C. (1983). Alpha‐1‐microglobulin: An indicator protein for renal tubular function. Journal of Clinical Pathology, 36(3), 253–259. 6186698 10.1136/jcp.36.3.253 PMC498194

